# Development of a new method based on unmodified gold nanoparticles and peptide nucleic acids for detecting bovine viral diarrhea virus-RNA

**DOI:** 10.1186/s13568-017-0432-z

**Published:** 2017-06-26

**Authors:** Maryam Askaravi, Seyedeh Elham Rezatofighi, Saadat Rastegarzadeh, Masoud Reza Seifi Abad Shapouri

**Affiliations:** 1 Department of Biology, Faculty of Science, Shahid Chamran University of Ahvaz, 6135743135 Ahvaz, Iran; 2 Department of Chemistry, Faculty of Science, Shahid Chamran University of Ahvaz, Ahvaz, Iran; 3 Department of Pathobiology, Faculty of Veterinary, Shahid Chamran University of Ahvaz, Ahvaz, Iran

**Keywords:** Gold nanoparticles, Detection, Bovine viral diarrhea virus, Peptide nucleic acid

## Abstract

A simple colorimetric assay is presented for detecting bovine viral diarrhea virus (BVDV)-RNA based on aggregation of gold nanoparticles (AuNPs) in the presence of charge-neutral peptide nucleic acids (PNA). Free charge-neutral PNA oligomers tended to be adsorbed onto AuNPs and act as a coagulant, whereas hybridizing complementary RNA with PNA disrupted PNA-induced AuNP aggregation, and the NPs remained stable. However, non-complementary RNA did not have this effect, and PNA induced aggregation of the AuNPs that resulted in a color change of the reaction from red to blue. The label-free colorimetric assay developed was estimated to have a 10.48 ng/reaction BVDV-RNA detection limit for the visual assay and 1.05 ng/reaction BVDV-RNA using a spectrophotometer. Diagnostic sensitivity and specificity for the assay was in accordance with real-time reverse transcriptase–polymerase chain reaction (RT-PCR) and nested RT-PCR results were 98 and 100%, respectively. Absorption of the 520/620 nm ratio was linear, along with an increase in the target RNA concentration of 1.64–52.4 ng/reaction (R^2^ = 0.992), which showed a linear correlation for the quantitative assay. This study established a rapid visual label and enzyme-free diagnostic assay for detecting BVDV that is applicable in any clinical laboratory.

## Introduction

Bovine viral diarrhea (BVD), an economically important disease of cattle, is caused by BVD virus (BVDV) a member of the genus *Pestivirus* of the *Flaviviridae* family (Ridpath and Passler 2016). Antigenic and nucleotide sequence differences of BVDV isolates distinguish two types of species, BVDV-1 and BVDV-2. Recently, a third putative species referred to ‘‘HoBi-like,’’ ‘‘BVDV-3’’ or ‘‘atypical pestiviruses’’ is reported (Cai et al. 2016; Bauermann et al. 2013). BVDV suppresses the immune system of an animal and makes it susceptible to other infections. It also reduces the herd productivity, milk yield, and reproductive efficiency of the cattle. BVDV is responsible for respiratory disorders, growth retardation, congenital defects, and increased mortality in cattle (Pinior et al. 2017; Houe 1999). BVD can be prevented by detecting and removing the infection source, in particular, in animals that may transfer the virus to uninfected animals (Pinior et al. 2017; Houe et al. 2006). Cattle with persistent infection (PI) of BVD often appear to be healthy; therefore, a sensitive and specific diagnostic test is essential. Improper diagnosis can be disastrous as it may indicate the removal of a valuable animal from the herd or retention of an infected calf in the herd. Several diagnostic tests such as antigen-capture enzyme-linked immunosorbent assay (AC-ELISA), immunofluorescence (IF), conventional reverse transcriptase–polymerase chain reaction (RT-PCR), and real time PCR are available for detection of BVDV; however, most of these tests require specialized and expensive devices that limits their application or use in local laboratories or in the field (Aebischer et al. 2014a, b).

Natural nucleic acids with some modifications can be used as biosensors in different fields of molecular diagnostics (Briones and Moreno 2012); however, this category of molecules introduces some limitations due to their features and backbone charge. To overcome these limitations, different families of nucleic acid analogs were developed (Briones and Moreno 2012). Peptide nucleic acid (PNA), a synthetic DNA analog, attains high biosensor sensitivity and specificity (Briones and Moreno 2012), and consists of repeating *N*-(2-aminoethyl)-glycine units linked by amide bonds, which attach purine and pyrimidine bases through methylene carbonyl linkages (Nielsen and Egholm 1999; Paulasova and Pellestor 2004). The unique physicochemical properties of PNA with neutral charge on its backbone enable it to bind strongly and specifically with complementary targets (RNA, DNA, or PNA) in a sequence dependent manner based on the base-pairing rules of Watson–Crick (Briones and Moreno 2012). The hybridization of PNA with DNA or RNA is highly specific, and base mismatches significantly decrease the thermal stability of PNA/DNA or PNA/RNA hetero-duplexes than DNA/DNA or RNA/DNA duplexes (Paulasova and Pellestor 2004). This high degree of segregation at single base level reveals that PNA probes that are shorter than the equivalent DNA or RNA probes could provide higher specificity; therefore, they are suitable for the development of PNA-based strategies in molecular diagnosis (Paulasova and Pellestor 2004). Interaction of PNA with RNA is independent of the RNA secondary structure (Paulasova and Pellestor 2004). Moreover, the use of low salt concentrations in hybridization buffer decreases the stability of the secondary structures of RNA; therefore, less accessible targets of RNA are exposed, and PNA probes can be hybridized easily with RNA (Lehtola et al. 2005). Notably, PNA is also resistant to the degradation by proteases and nucleases due to an artificial polyamide backbone; thus, the lifetime of PNAs is extended both in vitro and in vivo (Paulasova and Pellestor 2004; Zhang and Appella 2010).

**Scheme 1:**
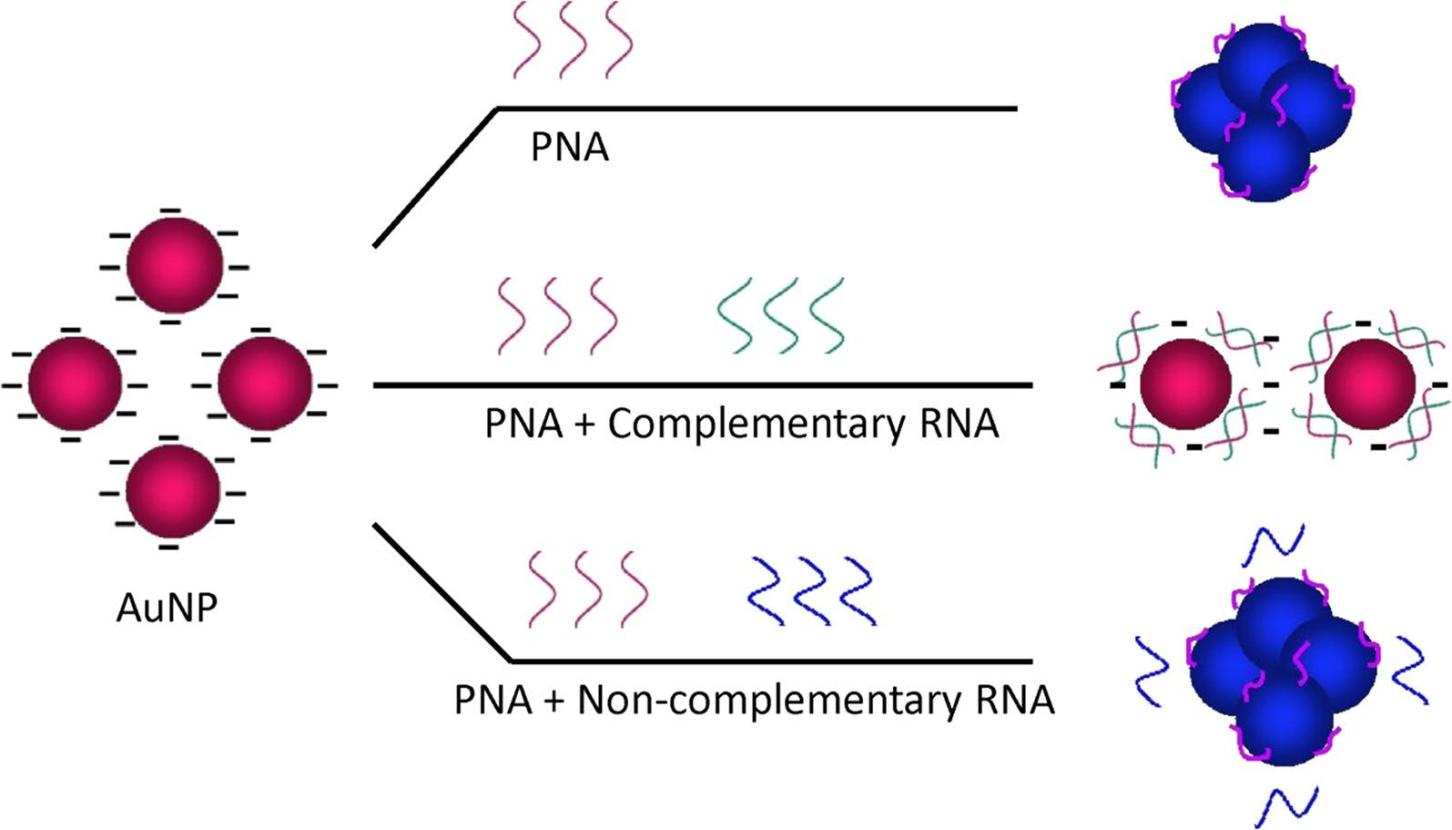
Schematic illustration of PNA-AuNP colorimetric detection assay. PNA immediately aggregates the AuNPs; however, hybridization of PNA with complementary RNA protects AuNP from aggregation, and the NPs remain stable. Non-complementary RNA did not hybridize with PNA; therefore, AuNP was aggregated in the presence of a charge-neutral PNA

Recently, nanoparticles are widely used in the diagnostic methods. In particular, gold nanoparticles (AuNPs) are extensively used in biosensor development due to their unique optical properties including distance-dependent color, high extinction coefficients, and fluorescence quenching ability (Zhang et al. 2012). Li and Rothberg (2004) found that AuNPs coated with citrate ions undergo different electrostatic reactions against single- and double-stranded oligonucleotides (ssDNA and dsDNA) when salt induced aggregation is performed. This property can be used for nucleic acid detection without covalent immobilization of DNA on the surface of AuNPs. Several methods have been developed based on AuNPs and PNA for detection of biomolecules, including those as explained by Endo et al. (2005) immobilized PNA on an Au-capped nanoparticle layer. Hybridization of PNA with its nucleic acid target led to thickness of the biomolecular layer and change in the optical properties of Au-capped NP layer. Other researchers have developed a specific PNA/DNA biosensor with immobilizing of PNA onto Au-magnetic nanoparticles. Interaction of PNA with a complementary DNA target was monitored by an external fluorescent molecule such as Rhodamine 6G (Pita et al. 2008). These methods are very sensitive; however, they are sophisticated and cannot be performed in a clinical field laboratory. Su and Kanjanawarut (2009) found that mixed-base PNA oligomers can induce immediate aggregation of AuNPs; whereas, hybridization of PNA to a complementary nucleic acid can greatly disrupt the aggregation. PNA molecules, when added to AuNPs due to neutral charge serve as a “coagulant” and induce particle aggregation in absence of salt, there by leading to a color change in AuNPs from red (with a specific surface plasmon peak at 520 nm) to dark purple (with the adsorption peak at 620 nm). In contrast, when PNA hybridizes to a complementary nucleic acid (DNA or RNA), the AuNPs solution remains stable. This phenomenon may occur when PNA-DNA or PNA-RNA is adsorbed on AuNPs, providing sufficient charge repulsion, required to disperse AuNPs using the negative charge of phosphate backbone on the DNA or RNA strands (Kanjanawarut and Su 2009). The aforementioned phenomenon can be used for detection of specific viral DNA or RNA (Scheme 1). In this study, we developed a rapid visual assay for detecting BVDV-RNA using PNA and unmodified AuNPs.

## Materials and methods

### AuNP synthesis

Citrate-stabilized AuNPs (∼13 nm) were synthesized by citrate reduction methods. In brief, 50 ml of a 0.01% tetrachloroauric acid (HAuCl_4_) solution was heated under vigorous stirring until boiling; then 600 μl of 1% sodium citrate was added quickly while heating was stopped and stirring was continued. After a few minutes, the color solutions changed from yellow to red. The AuNPs were then characterized by UV–Vis spectroscopy (GBC, Cintra 101, Australia) and transmission electron microscopy (TEM) (Zeiss Em10C, 80 kV, Germany).

### Reference isolates, BVDV archival isolates, field isolates, and other pathogens

Reference isolates of cpNADL (BVDV-1) and BVDV-2 (Razi strain) were provided by the Razi Vaccine and Serum Research Center (Hessarak, Iran). The strains were propagated in a Madin–Darby bovine kidney (MDBK) cell line. The reference samples were used as positive controls to develop and optimize the PNA-AuNP colorimetric detection assay.

Thirty blood samples from calves and adult bovines suspected of BVD were collected and subjected to RNA extraction. The observed clinical signs commonly include fever, respiratory disorders, diarrhea, reproductive dysfunctions and mucosal disease. Also, 36 positive BVDV archived isolates were collected in our laboratory, in the years of 2014–2016 from different farms in Khuzestan, Iran, and identified previously by RT-PCR were used in this study to evaluate the reaction specificity.

Some other bovine pathogens, such as bovine herpesvirus-1 (BoHV-1) (ATCCVR2112), foot-and-mouth disease virus (FMDV/O/2010/IRAN), bovine leukemia virus cultivated on fetal lamb kidney (BLV-FLK), *Mycobacterium bovis* (strain BCG), *Escherichia coli* O157:H7 (ATCC 43895), and *Pasteurella multocida* (ATCC12948) were used as negative controls.

### PNA design and synthesis

The probe sequence is directed against a conserved region of the 5′-untranslated region (5′UTR) of BVDV-1 and BVDV-2. 5′UTR sequences from 30 randomly chosen BVDV-1 and 15 randomly chosen BVDV-2 samples were retrieved from the National Center for Biotechnology Information (NCBI) Ref Seq database and aligned using ClustalX to identify the conserved region in the nucleotide sequences. The specificity of the probe was also checked using the available sequence data against the other pestiviruses as border disease virus (BDV) and classical swine fever virus (CSFV) to ensure no cross-reactions with the genome segments. PNA was synthesized by Panagene Co in Korea. The sequence and position according to the NADL genome sequence is presented in Table 1.

**Table 1:** PNA probe, primers, and target sequences used in PNA-AuNP colorimetric detection assay, multiplex RT-nPCR, and Real-time RT-PCR

Assay	Name	Sequence (5′ → 3′)	Reference
PNA-AuNP colorimetric detection assay	PNA^a^ Deleted target Mismatch target	GTACTCAGGGCTT AAAGCCAGTACGT AAGCGCAGTGTAC	This study This study This study
Multiplex RT-nested PCR	Exf Exr Bvdv1 Bvdv2 Ln	AAGATCCACCCTTATGA(A/G)GC AAGAAGCCATCATC(A/C)CCACA TGGAGATCTTTCACACAATAGC GGGAACCTAAGAACTAAATC GCTGTTTCACCCAGTT(A/G)TACAT	Gilbert et al. (1999) Gilbert et al. (1999) Gilbert et al. (1999) Gilbert et al. (1999) Gilbert et al. (1999)
Real-time RT-PCR	Pesti-3F Pesti-4R TQ-Pesti probe	CCTGAGTACAGGRTAGTCGTCA GGCCTCTGCAGCACCCTATCA FAM-TGCYAYGTGGACGAGGGCATGC-BHQ-1	Aebischer et al. (2014b) Aebischer et al. (2014b) Aebischer et al. (2014b)

^a^ Genome position: 186–198 of NADL-BVDV strain, GenBank: m31182.1

### RNA and DNA extraction

Viral RNA was isolated from MDBK infected with BVDV-NADL and BVDV-2 separately using the Bioneer viral RNA extraction kit (Korea) according to the manufacturer’s protocol. In addition, viral RNA was also isolated from 36 positive BVDV archived isolates and 30 field samples. RNA isolated from non-infected MDBK cells, Razi bovine kidney (RBK) cells, and FMDV were used as negative controls. The concentrations of the RNA extracts were measured using a NanoDrop (Thermo scientific, USA) at a wavelength of 260 nm.

DNA was extracted from *P. multocida*, *E. coli* O157:H7, as well as BoHV-1 and FLK-BLV, using a DNA extraction Kit (Bioneer, Korea) according to the manufacturer’s instructions. All extracted RNA and DNA were stored at −20 °C until used.

### PNA-AuNP colorimetric detection assay

Initially, to find the minimum concentration of PNA that caused aggregation of AuNPs, different concentrations of PNA were added to a fixed amount of AuNPs in each experiment. Color changes and absorption spectra of the AuNP solutions containing PNA were recorded.

Developed visual assays were performed in two steps of hybridization and detection. One microliter of RNA was added to a hybridization buffer containing PNA in final concentrations of 100 ρmol, 100 mM NaCl, 10 mM phosphate buffer (pH 7.2), and 0.1 mM EDTA for 10 min at 37 °C. Six microliter of hybridization reaction was added to 114 of AuNPs (20 nM). To improve the selectivity of the reaction and accelerate aggregation of AuNPs, NaCl (final concentration of 0.1 M) was added to the mixture. After 10 min incubation at room temperature, the color and absorption spectrum was recorded using a UV–Visible spectroscopy in a wavelength range of 500–640 nm. To analyze the spectral changes, an AuNP solution (red color) and a PNA-AuNP solution (blue color) were used as references for a comparison of the presence or absence of the RNA-targets.

### Multiplex RT-nested PCR and real-time RT-PCR

The multiplex RT-nPCR and real-time RT-PCR for detecting BVDV-1 and -2 were developed by Gilbert et al. (1999) and Aebischer et al. (2014b) respectively (Table 1), and these were used to compare with the PNA-AuNP colorimetric detection assay using the same templates on sensitivity.

### Analytical sensitivity and specificity of PNA-AuNP colorimetric detection assay

To determine the detection limit of developed method, viral RNA was extracted from the supernatant of the MDBK cells infected with cpNADL-BVDV or BVDV-2 and was quantified with a NanoDrop. Twofold serial dilutions were used on extracted RNA from 1.64 to 52.4 ng/μl and used for evaluation of the reaction specificity.

The specificity of the developed method was evaluated by testing other bovine pathogens, including BoHV-1, FMDV, BLV, *M. bovis*, *E. coli* O157:H7, and *P. multocida*. Non-infected MDBK cells, RBK cells, as well as mismatch, and deleted target DNA, were also analyzed by this assay (Table 1).

### Relative specificity and sensitivity of PNA-AuNP colorimetric detection assay

A total of 30 clinical blood samples and 36 archived samples were analyzed by the PNA-AuNP colorimetric detection assay as described above. The results of the assay were compared to the results of the multiplex RT-nPCR and real-time RT-PCR on the same templates.

## Results

### Optimization of PNA-AuNP colorimetric detection assay

**Fig. 1:**
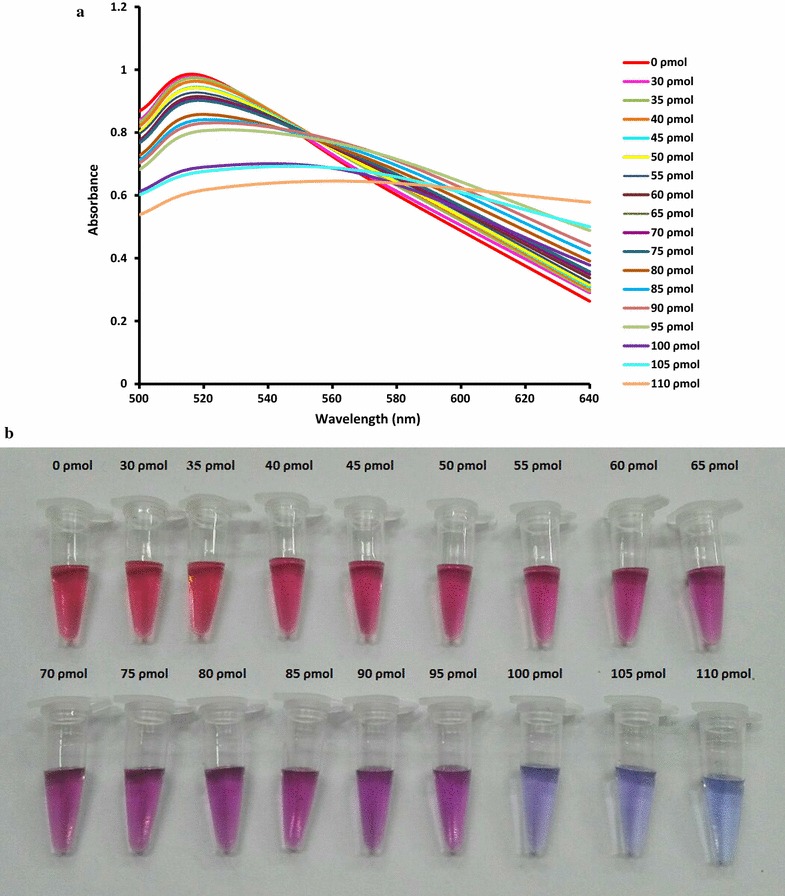
Optimization of PNA concentration required to aggregation of AuNP. **a** Absorption spectra of AuNP solutions containing different concentrations of PNA. **b** Visual colors of corresponding solutions

**Fig. 2:**
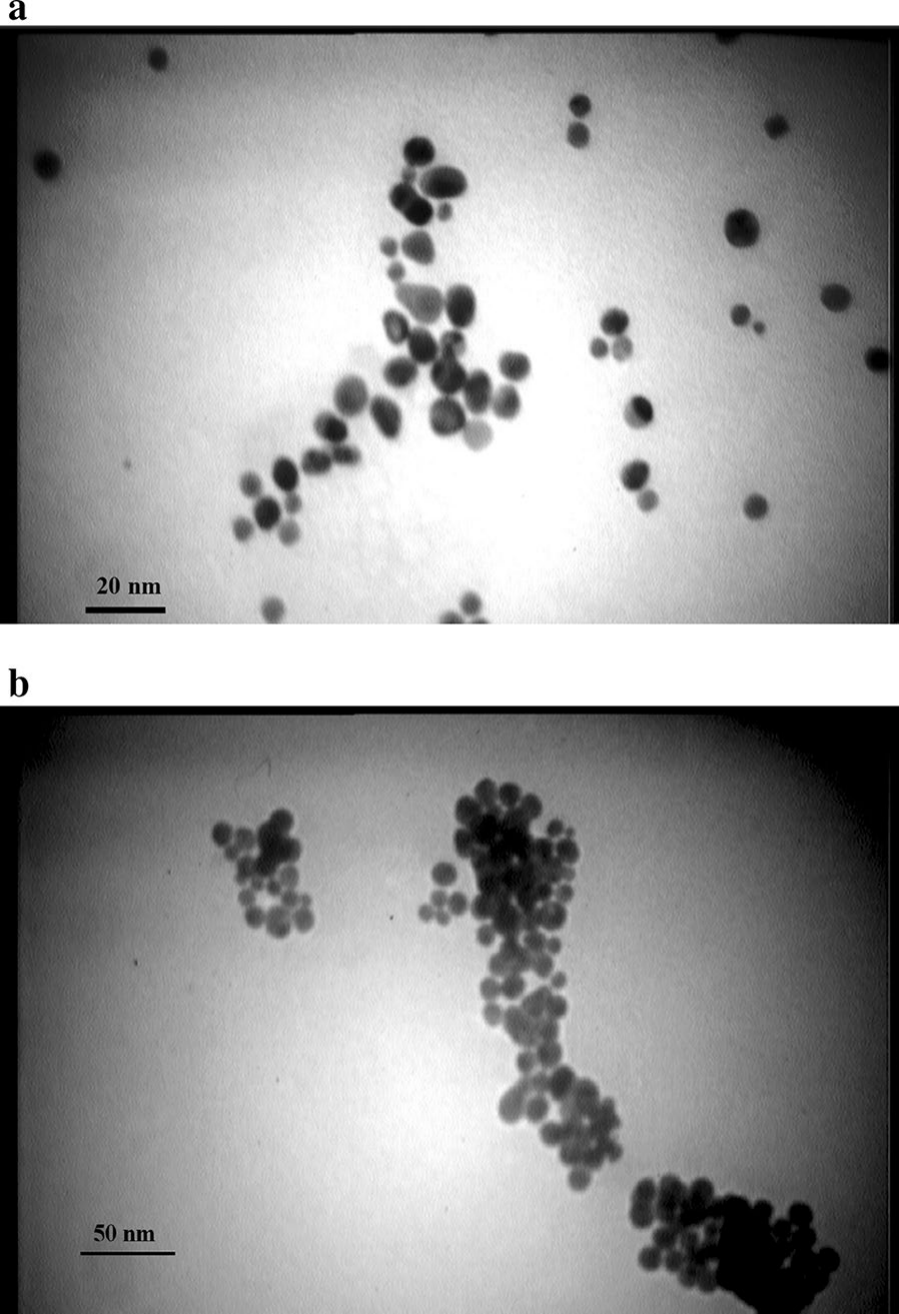
TEM images of AuNPs in the presence of positive and negative samples. RNA of positive sample inhibits PNA induced aggregation of AuNPs (**a**) while AuNPs are aggregated in presence of PNA and negative sample RNA (**b**)

To detect BVDV-RNA, a 13-mer PNA probe complementary to the 5′UTR of the BVDV-1 and BVDV-2 genomes was used. To obtain the best results, optimization of the PNA concentration is necessary, because very low PNA concentrations cannot induce AuNP aggregation in the absence of the target, leading to false positive results. On the other hand, high PNA concentrations induce AuNP aggregation in the presence of the low concentration of target, leading to false negative results. Various concentrations of the PNA probe were tested, and followed by increasing the PNA concentration in the reaction, the maximum absorbance at 520 nm was decreased, and a new peak was appealed at approximately 620 nm (Fig. 1). On the basis of visual analysis and according to the best signal-to-noise level, 100 ρmol of the PNA probe was the minimum concentration that induces gold aggregation in a 120 µl test solution and was suitable for the developed assay. As shown in Fig. 1, unmodified AuNPs showed an obvious absorption peak at 520 nm, while aggregated AuNPs by PNA had a blue shift to 620 nm. The PNA concentration-dependent absorption changes in the test reaction can be useful for the quantification of viral RNA (Joshi et al. 2013). The visual colors of reactions revealed that 10.48 ng/reaction of viral RNA is sufficient to inhibit the PNA-induced AuNPs aggregation (Fig. 2a). Below this concentration, the color of the reaction gradually tended toward purple (Fig. 2b).

After hybridization step AuNP was added to reaction and color changes were recorded visually and also by spectroscopy. In positive samples, no color change of AuNPs was observed, and λ_max_ of absorbance was at approximately 520 nm. This would be attributable to formation of PNA-RNA complementary complex and binding to AuNPs that can effectively protect AuNPs against PNA-induced aggregation. In contrast, in negative samples the color changed from red to blue and the surface plasmon peak shift from 520 nm to longer wavelengths of 600–620 nm, which shows evidence of the PNA-AuNPs interactions.

### Analytical specificity

**Fig. 3:**
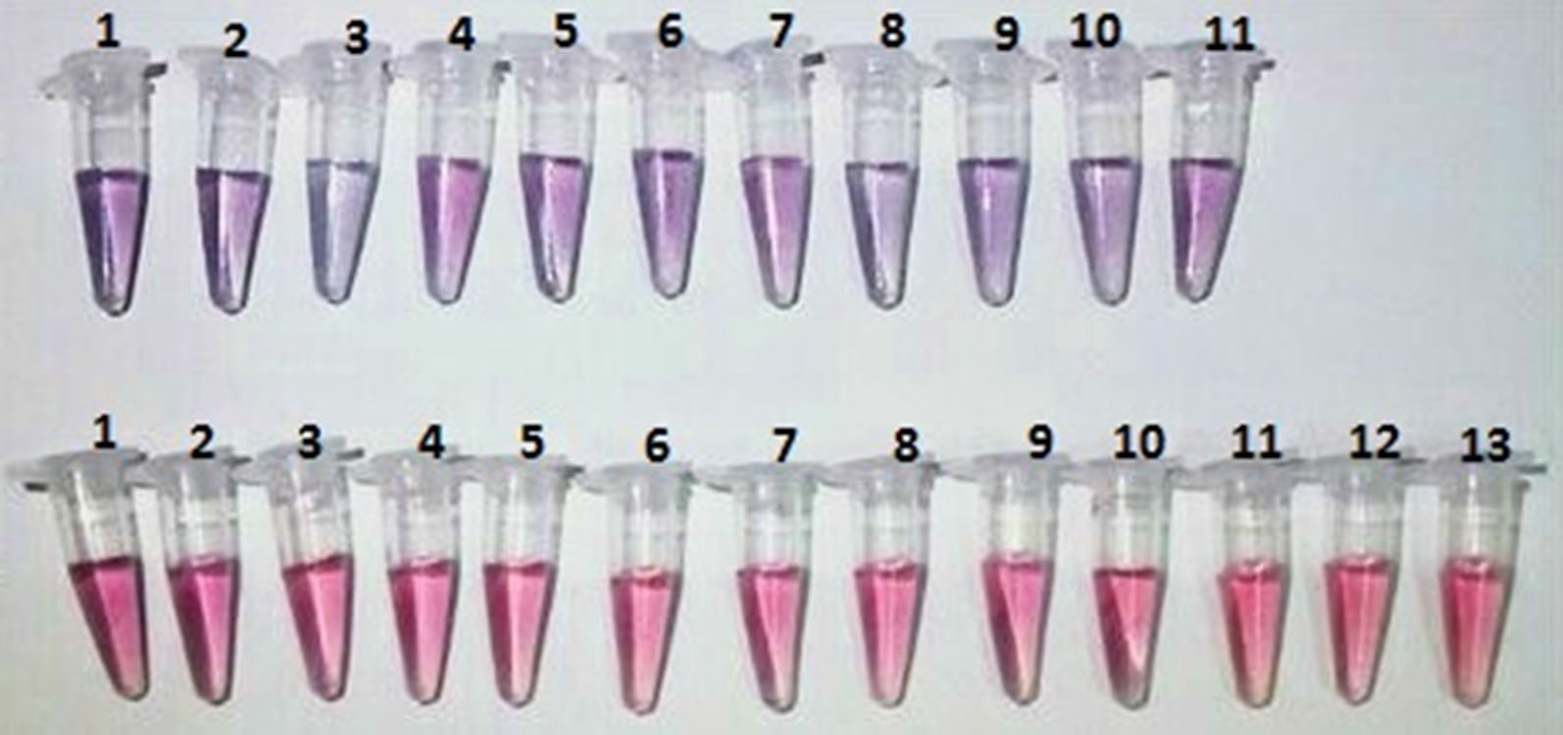
Visual observation of PNA-AuNP colorimetric assay in presence of negative and positive samples. *Upper row* is negative samples from *left to right*, *1* mismatched DNA target, *2* deleted DNA target, *3* FMDV, *4* BoHV, *5 P. multocida*, *6 E. coli* O157:H7, *7 M. bovis*, *8* BLV, and *9*–*11* negative field samples. *Lower row* is positive samples from *left to right*, *1* BVDV-NADL; *2* BVDV-2, *3*–*8* positive field samples, and *9*–*13* archived isolates

The PNA probe was designed based on the conserved sequence of 5′UTR. Sequence alignment showed that the probe is specific for BVDV-1 and -2 but not for the other species of pestiviruses (CSFV and BDV). The developed assay detected and matched DNA, cpNADL-BVDV, BVDV-2, whereas no cross-reactivity was observed with other bovine pathogens of FMDV, BoHV, *P. multocida*, *E. coli* O157:H7, *M. bovis*, and BLV, as well as RNA samples extracted from non-infected bovine cell lines, mismatch, and deleted target DNA. In positive samples the reaction color remained red; while in negative samples, the color shifted to blue (Fig. 3).

**Fig. 4:**
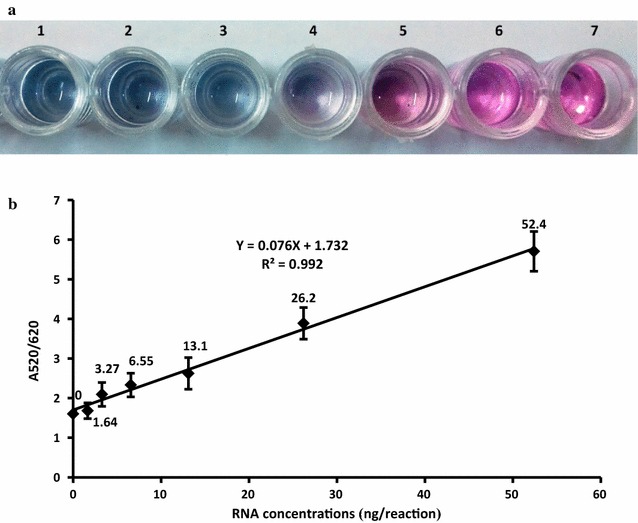
**a** Visual color changes of developed detection assay at different BVDV-RNA concentrations. Wells *1*–*7* contain 0, 1.64, 3.27, 6.55, 13.1, 26.2, and 52.4 ng/reaction of RNA respectively. **b** Plot of linear correlation (R^2^ = 0.992) of absorbance 520/620 nm ratio vs. different concentrations of viral RNA

To evaluate the analytical sensitivity of the newly developed BVDV assay, a twofold serial dilution of BVDV-RNA was used. As observed in Fig. 4a the visual detection limit for the viral RNA was gain 10.48 ng/reaction and below this concentration, the reaction color changes to blue. The absorption spectra and ratio of absorbance at 520 and 620 nm of dilutions were recorded. Spectral changes for the corresponding solutions determine the lower detection limit of 1.05 ng/reaction for spectrophotometer instrument. Figure 4b shows the linear dependence of the absorption of 520/620 nm ratio, along with an increasing of the concentration of target RNA in the range from 1.64 to 52.4 ng/reaction with a R^2^ value equal to 0.992. These results show that the presence of a small amount of target RNA can disrupt AuNP aggregation by PNA.

The detection limits of real time RT-PCR and RT-nPCR were gain 10.48 × 10^−5^ and 10.48 × 10^−4^ ng/reaction respectively. In comparison, the sensitivity of the developed assay was 5 and 4 log10 lower than real time RT-PCR, and RT-nPCR, respectively.

### Detection of BVDV from clinical samples

Thirty field samples suspected of being BVDV and 36 archived isolates were tested by PNA-AuNP colorimetric detection assay, and they were compared with those of RT-qPCR and RT-nPCR. Fifty samples were positive by the developed assay, whereas 51 samples were positive by two other assays. No sample was positive by colorimetric assay and negative by RT-qPCR and RT-nPCR. The developed assay showed diagnostic sensitivity of 98% and diagnostic specificity of 100%. The overall agreement between the new assay and two other assays was 98.5%.

## Discussion

In the present study, the color changes of AuNPs when facing a PNA-complementary RNA, and PNA/non-complementary RNA were used to develop a rapid, label-free visual assay for the detection and quantification of BVDV-RNA. The uncharged backbone of PNA makes PNA/DNA or PNA/RNA hybridization independent to salt concentration, ionic strength, and changes in pH. This characteristic significantly facilitates the PNA hybridization with nucleic acids and also a more than tenfold reduction in hybridization times can be achieved (Nielsen and Egholm 1999; Paulasova and Pellestor 2004). In this colorimetric assay PNA is used as a “coagulant” in the absence of salt. However, adding salt improves the selectivity, because it accelerates the aggregation of AuNPs in the absence of a target complementary to PNA (Kanjanawarut and Su 2009).

Analysis of designed probe showed the sequence homology between random selected 5′UTR of BVDV-1 and BVDV-2 isolates and the designed probe. The new developed assay will be particularly useful in detecting BVDV-1, the major type of BVDV circulating in the cattle farms of Iran. To develop some of the molecular assays, such as PCR or LAMP, need to be designed with at least two or four primers, respectively; while, because of inherent genetic heterogeneity of BVDV, the design of the number of universal primers that are capable of detecting all BVDV isolates remains a challenge. However, in the diagnostic methods based on PNA probes due to high specificity and the strong binding of PNA with complementary targets in a sequence dependent manner, the use of one probe is sufficient and therefore the finding of homolog sequences between BVDV isolates is easier. Also, it was reported that for the detection of mismatched targets, the selection of a shorter sequence is better than a longer sequence, and in this case the color and spectrum difference is more noticeable between fully complementary and single-mismatched targets (Kanjanawarut and Su 2009).

Su and Kanjanawarut (2009) reported that the degree of aggregation is directly related to the PNA concentration. However, PNA hybridization with the complementary RNA retains the stability of AuNPs, thereby preventing gold aggregation and color change. This phenomenon would be used for the quantification of BVDV-RNA. High values of linear correlations (R^2^ = 0.992) between RNA concentrations and with an absorbance of 520/620 nm show that this assay is capable of being normalized for virus quantification.

The detection limit for the recent developed assay was determined to be 10.48 or 1.05 ng/reaction and should be sufficient for clinical samples. However, further tests on clinical samples are required to fully determine the diagnostic value of this method. The new developed method harbored lower analytical sensitivity than the real-time RT-PCR and multiplex RT-nPCR assays. The primers of multiplex RT-nPCR, real-time RT-PCR and PNA probe for colorimetric detection assays used in this study detected the same 5′UTR region of BVDV. Lower analytical sensitivity is presumably due to a lack of replication of targets in the newly developed assay. However, the sensitivity of PNA-AuNP colorimetric detection assay was higher than reported for the unmodified AuNPs-based assay by our research group (Heidari et al. 2016). This can be attributed to a higher affinity of PNA-RNA hybridization than DNA–RNA hybridization (Kanjanawarut and Su 2009). Generally, AuNP colorimetric assays harbored a moderate sensitivity in the nanomolar range (Kanjanawarut and Su 2009), which is equivalent to the sensitivity of the standard PCR assay (Sato et al. 2003, 2005; Li and Rothberg 2004) therefore, this limitation should not overshadow the advantages of this method.

Despite the good diagnostic sensitivity and specificity of this developed method, one positive sample detected by real-time PCR and RT-nPCR was not detected by the new assay. The failure of the test can presumably be attributed to the low viral RNA molecules of this sample, which were below the detection limit of the developed assay. Joshi et al. have successfully used this method for detection, genotyping, and quantification of the viral RNA of the Newcastle disease virus. They reported that this assay could be appreciated for the molecular surveillance of viral diseases and clinical diagnostics (Joshi et al. 2013).

In conclusion, some advantages of the PNA-AuNP colorimetric detection assay include its acceptable sensitivity and specificity. In this method, viral RNA was detected directly, and there was no need to reverse transcription of RNA to cDNA. The new assay is much faster, simpler, and cost effective than polymerase chain reactions. It does not require the use of specific equipment thus it has potential applications in a clinical field laboratory. It does not need modification or conjugation of AuNPs or probes and the results are directly observed by visual detection. The lower sensitivity than nucleic acid replication methods could be a weak point of this method. Also, to prevent nonspecific reactions of samples with AuNPs, purified viral RNA should be used.

## Abbreviations

BVDV: bovine viral diarrhea virus
AuNP: gold nanoparticles
PNA: peptide nucleic acid
RT-PCR: reverse transcriptase–polymerase chain reaction
AC-ELISA: antigen-capture enzyme-linked immunosorbent assay
IF: immunofluorescence
PI: persistent infection
TEM: transmission electron microscopy
MDBK: Madin–Darby bovine kidney
BoHV-1: bovine herpesvirus-1
FMDV: foot-and-mouth disease virus
BLV-FLK: bovine leukemia virus-fetal lamb kidney
5′UTR: 5′-untranslated region
RBK: Razi bovine kidney
